# Photodynamic Therapy-Based Strategies Targeted at Cancer Stem Cells: A Scoping Review

**DOI:** 10.3390/cancers18071162

**Published:** 2026-04-03

**Authors:** Beatriz Serambeque, Inês Dias, Catarina Mestre, Carlos Miguel Marto, Maria Filomena Botelho, Maria João Carvalho, Mafalda Laranjo

**Affiliations:** 1 Univ Coimbra, Coimbra Institute for Clinical and Biomedical Research (iCBR) Area of Environment Genetics and Oncobiology (CIMAGO), Institute of Biophysics, Faculty of Medicine, 3000-548 Coimbra, Portugal; uc2023185299@student.uc.pt (I.D.); uc2016239230@student.uc.pt (C.M.); cmiguel.marto@uc.pt (C.M.M.); mfbotelho@fmed.uc.pt (M.F.B.); mjcarvalho@fmed.uc.pt (M.J.C.); 2 Univ Coimbra, Center for Innovative Biomedicine and Biotechnology (CIBB), 3000-548 Coimbra, Portugal; 3 Clinical Academic Centre of Coimbra (CACC), 3004-561 Coimbra, Portugal; 4 Univ Coimbra, Institute of Experimental Pathology, Faculty of Medicine, 3000-548 Coimbra, Portugal; 5 Univ Coimbra, Institute of Integrated Clinical Practice and Laboratory for Evidence-Based Sciences and Precision Dentistry, 3000-075 Coimbra, Portugal; 6 Univ Coimbra, Centre for Mechanical Engineering, Materials and Processes (CEMMPRE), Advanced Production and Intelligent Systems (ARISE), 3030-788 Coimbra, Portugal; 7 Gynaecology Service, Department of Gynaecology, Obstetrics, Reproduction and Neonatology, Unidade Local de Saúde de Coimbra, 3004-561 Coimbra, Portugal; 8 Univ Coimbra, Universitary Clinic of Gynecology, Faculty of Medicine, 3004-561 Coimbra, Portugal

**Keywords:** cancer stem cells, photodynamic therapy, photosensitizer, stemness, targeted therapies

## Abstract

A major challenge in cancer treatment is the presence of cancer stem cells (CSC), which play a significant role in therapeutic resistance, metastatic progression, and tumor relapse, highlighting their importance as therapeutic targets. Photodynamic therapy (PDT) can be a valuable strategy for targeting CSC by increasing reactive oxygen species via light-activated photosensitizers, thereby counteracting CSC features of resistance. Therefore, this manuscript reviews the existing evidence on PDT-based approaches targeted at CSC. The literature search was conducted using the Medline (through PubMed), Web of Science, and Embase databases. The inclusion criteria were in vitro, in vivo, and clinical studies addressing PDT-based therapeutic strategies targeting CSC. A total of 37 publications were included in this review. From this analysis, we identified several studies with CSC-associated targets, delivery of PDT systems to CSC, novel nanoplatforms that disrupted the CSC microenvironment, and combined strategies to enhance PDT efficacy. The existing strategies have shown success in reducing CSC populations across different cancers by decreasing viability and tumor growth, as well as limiting stemness properties. However, some technical and biological constraints can limit translation, and, to the best of our knowledge, no clinical studies have been reported.

## Simple Summary

One of the biggest challenges in cancer treatment is a small group of cells called cancer stem cells, which are often responsible for therapy failure, cancer spread, and recurrence after treatment. The goal of this review is to examine strategies for targeting cancer stem cells using photodynamic therapy. Photodynamic therapy, which uses light-activated compounds to generate harmful molecules within cancer cells, has been investigated as a treatment for cancer, thereby destroying cancer stem cells. The findings highlight several promising approaches, including the use of advanced nanoparticles that can impair or eliminate these cell populations. While many strategies have been studied, both in vitro and in vivo, to our knowledge, no clinical studies have been reported.

## 1. Introduction

Cancer remains a significant global challenge, and one of the key contributors to anticancer therapy failure is the presence of cancer stem cells (CSC), a subpopulation of tumor cells that enable self-renewal, differentiation, and tumor initiation [1,2,3,4]. Their quiescent behavior, along with efficient antioxidant defenses, enables CSC to survive therapy, develop resistance, and recur [5]. These characteristics position CSC as a potential therapeutic target for the development of more effective anticancer treatments [6].

CSC can preserve embryonic self-renewal pathways, including Wnt, Notch, Hedgehog, JAK/STAT, and PI3K/AKT, which maintain their stem-like phenotype. Although these pathways are used by normal stem cells, their dysregulation in cancer supports CSC uncontrolled proliferation and survival [5,7]. Furthermore, CSC use both glycolysis and oxidative phosphorylation to meet energy demands while limiting the accumulation of reactive oxygen species (ROS), thereby reinforcing their antioxidant capacity [8,9]. Moreover, hypoxia plays a central role in maintaining CSC characteristics by stabilizing hypoxia-inducible factors (HIF-1α and HIF-2α), which will activate genes that promote stemness (*OCT4*, *SOX2*, and *NANOG*) [10]. In hypoxic circumstances, the expression of these genes can be involved in processes such as epithelial-to-mesenchymal transition (EMT) [11], further supporting invasiveness and resistance to programmed cell death [12].

Conventional therapeutic modalities often fail to eliminate CSC due to several intrinsic and microenvironment-mediated resistance mechanisms. CSC typically proliferate slowly, exhibit a robust deoxyribonucleic acid (DNA) repair mechanism, and maintain low ROS levels [13]. These ROS protection mechanisms that CSC may exhibit [14] can require therapies that produce higher levels of ROS to promote CSC differentiation [15] and, ultimately, enable CSC elimination.

Photodynamic therapy (PDT) is an anticancer approach that can generate higher levels of localized ROS upon activation of photosensitizers by light. In addition to the exogenous ROS produced during PDT, this modality can increase ROS production in CSC by altering metabolism or by inducing stress in organelles, such as the endoplasmic reticulum or mitochondria. The accumulation of photosensitizers into this cellular machinery can explain the effects of PDT on CSC, namely the induction of oxidative stress that damages DNA, disrupts organelle function, and triggers cell death [16]. Furthermore, there is evidence that PDT can induce necrosis in MCF7 chemoresistant cells, resulting from increased ROS production [17]. These mechanisms, along with CSC-targeted strategies, can enhance PDT’s potential to specifically eliminate CSC-enriched populations, thereby reducing the risk of tumor recurrence.

Given the potential of PDT, this scoping review aims to comprehensively map the research on PDT strategies targeting CSC, particularly describing how CSC respond to targeted PDT-based modalities, identifying molecular targets within these populations, and highlighting gaps that justify further investigation and may currently limit translation.

## 2. Materials and Methods

This review was conducted following the methodology proposed by Arksey and O’Malley [18] and the “Preferred Reporting Items for Systematic Reviews and Meta-Analysis, extension for Scoping Reviews (PRISMA-ScR)” guidelines [19].

### 2.1. Review Question

A review question was structured according to the Population, Concept, and Context model [20]: “What are the therapeutic strategies based on PDT targeted at CSC?”. A secondary research question was also formulated: “What are the specific CSC targets used to guide therapy?”.

### 2.2. Literature Search

The literature search was conducted in the Medline (via PubMed), Web of Science (all databases), and Embase databases on 4 February 2025. The PubMed search strategy was (“Photochemotherapy” [Mesh] OR Photochemotherap* OR chemophototherap* OR “hematoporphyrin photoradiation” OR “photo-activated chemotherapy” OR “photo-chemotherapy” OR “photoactivated chemotherapy” OR “Photodynamic Therap*” OR “Therap*, Photodynamic” OR “Red Light PDT” OR “Light PDT, Red” OR “PDT, Red Light”) AND (“Neoplastic Stem Cells” [Mesh] OR “Neoplastic Stem Cell*” OR “Cell*, Neoplastic Stem” OR “Stem Cell*, Neoplastic” OR “Tumor Stem Cell*” OR “Cell*, Tumor Stem” OR “Stem Cell*, Tumor” OR “Tumor-Initiating Cell*” OR “Tumor Initiating Cell*” OR “Cell*, Tumor Initiating” OR “Initiating Cell*, Tumor” OR “Cancer Stem Cell*” OR “Cell*, Cancer Stem” OR “Stem Cell*, Cancer” OR “Neoplastic Colony-Forming Unit*” OR “Colony-Forming Unit*, Neoplastic” OR “Neoplastic Colony Forming Unit*” OR “Unit*, Neoplastic Colony-Forming” OR “Colony Forming Unit*, Neoplastic”). In Web of Science, the search strategy was (Photochemotherap* OR chemophototherap* OR “hematoporphyrin photoradiation” OR “photo-activated chemotherapy” OR “photo-chemotherapy” OR “photoactivated chemotherapy” OR “Photodynamic Therap*” OR “Therap*, Photodynamic” OR “Red Light PDT” OR “Light PDT, Red” OR “PDT, Red Light”) AND (“Neoplastic Stem Cell*” OR “Cell*, Neoplastic Stem” OR “Stem Cell*, Neoplastic” OR “Tumor Stem Cell*” OR “Cell*, Tumor Stem” OR “Stem Cell*, Tumor” OR “Tumor-Initiating Cell*” OR “Tumor Initiating Cell*” OR “Cell*, Tumor Initiating” OR “Initiating Cell*, Tumor” OR “Cancer Stem Cell*” OR “Cell*, Cancer Stem” OR “Stem Cell*, Cancer” OR “Neoplastic Colony-Forming Unit*” OR “Colony-Forming Unit*, Neoplastic” OR “Neoplastic Colony Forming Unit*” OR “Unit*, Neoplastic Colony-Forming” OR “Colony Forming Unit*, Neoplastic”). Awarded Grant OR Book OR Abstract OR Dissertation Thesis OR Meeting OR Editorial Material OR Retracted Publication OR Patent were excluded in the search. Regarding Embase, the search approach included (‘photochemotherapy’/exp OR ‘chemophototherap*’ OR ‘hematoporphyrin photoradiation’ OR ‘photo-activated chemotherapy’ OR ‘photo-chemotherapy’ OR ‘photoactivated chemotherapy’ OR ‘photochemoterap*’ OR ‘therap*, photodynamic’ OR ‘photodynamic therap*’ OR ‘red light pdt’ OR ‘light red, pdt’ OR ‘pdt, light red’) AND (‘cancer stem cell’/exp OR ‘cancer stem cell*’ OR ‘stem cell, tumour’ OR ‘tumor-initiating cell*’ OR ‘neoplastic stem cell*’ OR ‘cell*, neoplastic stem’ OR ‘stem cell*, neoplastic’ OR ‘tumor stem cell*’ OR ‘cell*, tumor stem’ OR ‘stem cell*, tumor’ OR ‘tumor initiating cell*’ OR ‘cell*, tumor initiating’ OR ‘initiating cell*, tumor’ OR ‘cell*, cancer stem’ OR ‘stem cell*, cancer’ OR ‘neoplastic colony-forming unit*’ OR ‘colony-forming unit*, neoplastic’ OR ‘neoplastic colony forming unit*’ OR ‘unit*, neoplastic colony-forming’ OR ‘colony forming unit*, neoplastic’) AND ([article]/lim OR [article in press]/lim OR [data papers]/lim OR [letter]/lim OR [review]/lim). A language filter was used, and articles in English, Portuguese, Spanish, or French were considered in all searches. No temporal restrictions were applied.

### 2.3. Study Selection

Database search results were imported into online EndNote, and duplicates were removed. The results were first screened by title and abstract, and later by full text. The eligibility criteria included original studies (in vitro, in vivo, or clinical) that addressed PDT-based therapeutic strategies targeting CSC. Articles addressing the effect of PDT on CSC without a targeted approach or other therapeutic modalities were excluded.

### 2.4. Data Extraction

The data were collected and summarized in tables. The data collected includes the disease and the source of CSC; the target and targeting strategy; information about PDT (photosensitizer used, drug-light interval (DLI), light source, and fluence/energy); and the methods used, along with the main results. Additionally, a narrative description was provided. This review follows the “PRISMA-ScR Checklist” review protocol [19].

## 3. Results and Discussion

**Figure 1:**
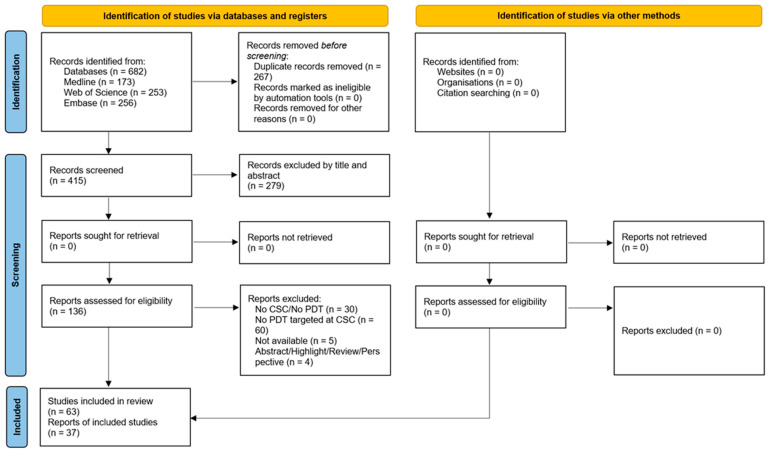
Flow diagram of the selection of evidence sources included and reasons for exclusion.

The search for targeting approaches to eliminate CSC using PDT-based methods has been extensively documented across in vitro and in vivo studies. Figure 1 details the number of articles resulting from the search, articles screened and included, along with the reasons for exclusion at each phase.

### 3.1. PDT-Based Approaches Targeted at CSC-Associated Markers

Studies addressing PDT-based approaches targeting CSC-associated markers are summarized in Table 1.

Targeting aldehyde dehydrogenase (ALDH), an enzyme frequently overexpressed in CSC, can be a promising strategy for selectively eliminating these populations [21]. ALDH is involved in detoxification and has been associated with resistance to chemotherapy and radiotherapy. However, its effect on PDT sensitivity, particularly with the photosensitizer meso-tetraphenyl chlorine disulfonate (TPCS_2_a), was previously unclear. To clarify this, HT-29 human colon cancer cells expressing CD133 were sorted into ALDH^bright^, ALDH^dim^, and unsorted populations using the ALDEFLUOR assay. Each group received conventional chemotherapeutic agents, ionizing radiation, and TPCS_2_a-mediated PDT. The results showed no significant differences in sensitivity to TPCS_2_a-PDT between ALDH^bright^ and ALDH^dim^ cells. However, ALDH^bright^ cells exhibited resistance to ionizing radiation, indicating that ALDH activity contributes to radioresistance but does not affect the efficacy of TPCS_2_a-mediated PDT [22]. A recent strategy was also developed to target ALDH^high^ CSC by selectively enhancing photosensitizer accumulation. SCHO, a novel compound comprising a thionylated coumarin core and N-ethyl-4-(aminomethyl)benzaldehyde, was designed as an ALDH substrate. The results indicated that SCHO is efficiently metabolized in ALDH^high^ MDA-MB-231 breast cancer cells, resulting in selective intracellular retention. Importantly, SCHO avoids ABC transporter-mediated efflux, reinforcing its specificity for ALDH-expressing cells. Upon light activation, SCHO-based PDT induced both apoptosis and pyroptosis while reducing CSC-associated markers OCT4, Nanog, and SOX2 [23].

Cancer cell populations exhibiting a stem-like profile have been shown to have elevated tissue factor (TF) levels, which can play a significant role in the tumor-initiating capacity of these cells. In addition, TF expressed by tumor cells was suggested to contribute to the regulation of CSC behavior, influencing their survival, proliferation, and metastatic potential [24]. Nevertheless, a negative correlation between TF expression and CSC activity was observed in colorectal CSC, suggesting that TF may play a controversial role in CSC [25]. TF expression was confirmed in CSC-enriched populations of breast, lung, and ovarian models, and TF-targeted photoimmunotherapy using a factor VII-conjugated SnCe6 eradicated CD133^+^ CSC in H460 and A549 lung cancer cells, as well as eliminating non-CSC. To explore the mechanisms of action, CD133^+^ H460 CSC subjected to PDT showed apoptosis and necrosis, with evidence of membrane damage, whereas untreated cells showed no signs of cell death [26].

Another CSC-associated marker addressed is the leucine-rich repeat-containing G-protein-coupled receptor 5 (Lgr5). Lgr5-expressing cells are considered tumor-initiating cells in colorectal cancer, positioning them as potential therapeutic targets. However, as Lgr5 is also expressed in intestinal normal stem cells, achieving targeting specificity remains challenging. To overcome this limitation, researchers evaluated a PDT-based modality for the selective elimination of colon CSC to inhibit polyp formation. The hypothesis was that CSC are located deeper within colon tissue and that modulating PDT penetration could selectively target these cells while preserving normal stem cells. The study employed a strain of transgenic mice (Lgr5-EGFP-IRES-creERT2 knock-in mice) predisposed to colon polyps and to the development of precancerous lesions. The photodynamic treatment resulted in CSC elimination, significantly reduced colon polyps, and preserved normal stem cells [27].

**Table 1:** Studies addressing PDT-based approaches targeted at CSC-specific markers.

Ref.	Model	Disease/Source of CSC	Target/Targeting Strategy	Photodynamic Approach	Methodology for Assessing the Results	Main Results
[22]	In vitro	Human colon cancer HT-29 cells	ALDH TPCS_2a_	PS: TPCS_2a_ Concentration: 0.4 µg/mL DLI: 18 h Wavelength: 435 nm Light source: blue light Energy: 9.6 mW/cm^2^	Clonogenic assay	ALDH^bright^ HT-29 cells are significantly less sensitive to IR.
[23]	In vitro	Human Breast cancer MDA-MB-231cells	ALDH SCHO	PS: SCHO Concentration: 50 µM DLI: 2 h Light source: white light Energy: 25 mW/cm^2^ Treatment time: 10 min	ROS levels assay LDH activity CD44 and CD24 expression RT-PCR Cellular uptake	CSC characteristics are decreased after treatment
[26]	In vitro	Human Epithelial lung cancer A549 cells	CD133 CD-133 targeted with AuNP-PEG-AlPcS_4_Cl-Ab	PS: AlPcS_4_Cl Concentration: 20 µM DLI: 4 h Wavelength: 673.2 nm Light source: semiconductor diode laser Energy: 10 J/cm^2^	Morphology, LDH cytotoxicity, ATP proliferation, trypan blue viability, and annexin VPI	Localization in the cytoplasm and nuclei of cells due to the anti-CD-133 antibody biomarker
[27]	In vitro	Mouse colon adenocarcinoma Lgr5-EGFP-IRES-creERT2 polyps	Lgr5 Rose Bengal	PS: RB Concentration: 100 nM DLI: 4 h Wavelength: 473 nm Light source: blue diode light Energy: 7 mW/cm^2^	Cell death In vivo imaging Histological and gene expression Immunohistochemistry	Inhibited tumor growth and reduced the number of polyps
	In vivo			PS: RB Concentration: 50 nM (0.75 mL/kg) DLI: 4 h Wavelength: 473 nm Light source: blue diode laser Energy: 22–25 mW/cm^2^ Treatment time: 2 min Treatment duration: twice a week for 7 weeks		

**Legend:** ALDH—Aldehyde dehydrogenase; ATP—Adenosine TriPhosphate; DLI—drug-light interval; IR—Infrared; LDH—lactate dehydrogenase; ROS—Reactive oxygen species; RT-PCR—reverse transcription-polymerase chain reaction.

Enzyme-targeted PDT, particularly ALDH-related pathways, emerges as a promising strategy. While ALDH overexpression is widely associated with therapy resistance [28], its role in PDT responsiveness appears to be context-dependent. In colorectal CSC models, ALDH^bright^ cells did not exhibit altered sensitivity to TPCS_2_a-PDT compared with ALDH^dim^ populations despite resistance to ionizing radiation [22]. In contrast, the use of molecules such as SCHO demonstrates that ALDH enzymatic activity can be exploited for selective PDT delivery. SCHO effectively accumulated in ALDH^high^ breast CSC and triggered both apoptotic and pyroptotic pathways, suggesting that enzyme-activated photosensitizers may outperform conventional PDT agents in CSC-rich environments [23]. Regarding other marker-based strategies, such as targeting TF [27], variable results suggest that although TF-targeted PDT can be highly effective in certain tumor types, its applicability may require tumor-specific validation rather than assuming universal overexpression across CSC populations. Despite using different approaches, PDT-induced cell death frequently involves apoptosis/necrosis or immunogenic pathways such as pyroptosis, which may further disrupt the CSC-driven tumor microenvironment. On the other hand, the contrasting ALDH findings suggest that ALDH is not a universal predictor of PDT response. Nevertheless, ALDH-targeted PDT approaches appear particularly promising, as they have shown high selectivity for CSC, the ability to overcome ABC transporter-mediated drug efflux, induction of multiple cell death pathways, and downregulation of stemness markers.

### 3.2. Microenvironment-Responsive PDT Systems

This section summarizes PDT approaches designed to be CSC microenvironment-responsive. The main features and findings of these studies are outlined in Table 2.

Nanoparticles formed by the self-assembly of a simple amino-functionalized porphyrin (m-TAPP) with a short peptide (Fmoc-L3-OMe) were designed to selectively target CSC by exploiting their elevated ribosomal content. These nanoparticles were designed to respond to the acidic tumor microenvironment by acquiring a positive surface charge, which facilitated selective interaction with negatively charged ribosomal structures and nucleic acids within CSC. Given the high ribosome content and rapid replication rate of CSC, the researchers hypothesized that these cells would be particularly susceptible to positively charged small molecules, such as m-TAPP and their nanoparticle assemblies. To evaluate CSC targeting, the expression of key stemness markers, CD44 and CD133, was assessed in tumor tissues. The results showed that treatment with m-TAPP or nanoparticles, even without light activation, led to a modest reduction in CD44 expression, suggesting inherent CSC affinity. Interestingly, PDT-treated tumors exhibited increased expression of CD44 and CD133 despite significant cancer cell apoptosis. This paradoxical upregulation was attributed to ROS generated during PDT, which may stimulate residual CSC to produce more CD44 protein [29]. In contrast, other experiments showed that CSC marker expression was significantly reduced following PDT. Ribosome damage was confirmed, which showed a marked decrease in functional ribosomes post-treatment. This supported a positive correlation between ribosome disruption and reduction in CSC. Further analysis of stemness-associated genes *OCT4*, *NANOG*, and *SOX2* revealed substantial downregulation in nanoparticle-treated cells under light exposure. Finally, in vitro wound-healing assays demonstrated potent inhibition of cancer cell migration following PDT. In vivo, using a 4T1 mouse lung metastasis model, the same treatment reduced metastatic spread, highlighting the therapeutic potential of this strategy in both primary tumor control and metastasis prevention [29]. Also, in breast cancer, dualistic strategies have been investigated. A region-specific PROTAC-based nanoplatform (PGDAT@N) has been developed that integrates both ROS-activatable and hypoxia-responsive PROTAC prodrugs. The nanoplatform incorporated BRD4-targeting PROTACs (ARV771 derivatives) with dual responsiveness: a thioketal linkage for ROS-triggered activation under normoxic conditions and a nitrobenzyl group for hypoxia-responsive activation. Photosensitizer pyropheophorbide a (PPa) was integrated into the polymer backbone to enable PDT upon 671 nm laser irradiation, while an MMP-2-cleavable PEG facilitated tumor-specific activation. Upon internalization, the acidic pH of the intracellular space activates the photo-responsive component, thereby generating localized ROS through PDT. This ROS burst facilitates the release of the active PROTAC within normoxic tumor regions. Simultaneously, the system addresses the hypoxic niches typically occupied by CSC. In these zones, a hypoxia-responsive PROTAC prodrug is selectively activated by nitroreductase, an enzyme overexpressed in hypoxic cancer cells, ensuring BRD4 degradation even in low-oxygen environments. This dual activation mechanism enables comprehensive targeting of both normoxic tumor cells and hypoxic CSC, effectively addressing spatial and metabolic heterogeneity within tumors. In vitro, ROS generation was quantified using DCFH-DA, and BRD4 degradation was confirmed via Western blot. Acidic conditions (pH < 6.2) significantly enhanced PDT activation, resulting in an over sevenfold increase in ROS production. Cell viability assays demonstrated synergistic cytotoxicity resulting from the combined use of PDT and PROTAC release. In vivo, the nanoplatform was evaluated in MDA-MB-231 and HN30 xenograft models. Treatment resulted in marked tumor regression over 14 days, accompanied by increased apoptosis and necrosis in tumor tissue. Hypoxia-responsive activation further suppressed CSC markers, including CDK4 and CDK6, while upregulating p21. A histological analysis of the major organs revealed no significant toxicity [30].

The reviewed strategies demonstrate that CSC-targeted PDT can be achieved either by exploiting intrinsic CSC features, such as elevated ribosomal content and acidic microenvironments, or by designing systems that adapt to metabolic heterogeneity within tumors. m-TAPP/peptide nanoparticles selectively interact with ribosome-rich CSC, leading to ribosomal disruption, suppression of stemness genes, and reduced migration [29]. However, some models showed upregulation of CSC markers after PDT, likely reflecting ROS-induced stress responses. In contrast, the dual-responsive PGDAT@N platform integrates PDT with BRD4-targeting PROTACs that are activated under both normoxic and hypoxic conditions [30], thereby enabling comprehensive elimination of tumor cells, including hypoxic CSC niches that are typically resistant to therapy. In these studies, convergence was observed in the ability of PDT-based nanotechnologies to generate ROS, induce apoptosis or necrosis, and downregulate stemness-associated pathways. Overall, platforms like PGDAT@N appear more promising due to their capacity to address intratumoral heterogeneity, sustain suppression of stemness regulators, considering hypoxic regions, and demonstrate in vivo efficacy.

### 3.3. Delivery Systems for CSC-Targeted PDT

This section reviews nanoplatforms specifically engineered to enhance the delivery of photosensitizers and therapeutic agents to CSC. The studies included in this category are summarized in Table 3.

#### 3.3.1. Nanoparticles

The therapeutic potential of PDT in melanoma CSC was investigated using a nanoconjugate composed of Sulphonated Aluminum Phthalocyanine Chloride (AlPcS_4_Cl) and gold nanoparticles to target CD133^+^ CSC. Melanoma CSC were isolated using magnetic-activated cell sorting with CD133 microbeads. The nanoconjugate significantly enhanced PDT-induced cytotoxicity in CSC compared to the photosensitizer per se, increasing the proportion of apoptotic CSC, and elevating caspase-3 and P53 expression [31]. In another study, CSC were isolated from the A375 melanoma cell line using CD133 sorting and subsequently characterized for CD133 and CD20 expression. The isolated CSC were then incubated with the same nanoconjugate. After PDT, an increased lactate dehydrogenase release indicated cytotoxicity and membrane damage. Additionally, ATP assays demonstrated a substantial reduction in cellular proliferation, and trypan blue exclusion tests confirmed decreased viability of melanoma CSC [32]. In the case of lung CSC, a side population from the A549 cell line with a CD133^+^/CD44^+^/CD56^+^ phenotype demonstrated pronounced intracellular localization of the nanoconjugate in peri-nuclear regions and the cytoplasm. After treatment, lung CSC exhibited morphological changes, including cellular shrinkage, chromatin condensation, membrane blebbing, and cytoplasmic vacuole formation, which are indicative of apoptosis. Subsequent assays revealed significantly increased cytotoxicity (approximately 97%) and a cell viability of about 30% [33].

A multifunctional nanoplatform for breast cancer therapy was developed to co-deliver salinomycin (SAL), a CSC-selective agent, Ce6, and vitamin E acetate, forming SAL/Ce6@kVE nanoparticles [34]. SAL has shown substantial efficacy against breast CSC compared with paclitaxel, a widely used chemotherapeutic drug, with studies reporting an over 100-fold increase in potency. The mechanism of SAL involves induction of apoptosis in mammospheres, associated with downregulation of anti-apoptotic proteins, including Bcl-2. SAL also reduces the migratory capacity of these cells, which correlates with decreased expression of oncogenic and EMT-related markers, such as c-Myc and Snail. Multiple studies have confirmed SAL’s ability to reduce the CSC population in various models, including suppression of CSC-associated genes [35]. The nanoparticles were synthesized in aqueous media and characterized for their physicochemical properties, drug-loading efficiency, and ROS generation upon light activation. Therapeutic efficacy was evaluated in vitro using breast cancer cell lines (MDA-MB-231 and MCF-7) and CSC-enriched mammospheres. Photodynamic treatment significantly reduced cell viability and induced apoptosis and necrosis, demonstrating a synergistic cytotoxic effect. Mammosphere formation assays showed a pronounced reduction in CSC self-renewal capacity, dependent on nanoparticle concentration. In vivo, zebrafish embryo xenograft models were employed to assess tumor development using the transgenic Tg(7xTCFX.lasiam:EGFP) and Tg(hsa.cox8a:mls-EGFP) cell lines, and to investigate modulation of molecular pathways. PDT effectively suppressed tumor growth and disrupted the Wnt/β-catenin signaling pathway, a key regulator of CSC maintenance and tumor progression [34].

**Table 3:** Studies addressing delivery systems for CSC-targeted PDT.

Ref.	Model	Disease/Source of CSCs	Target/Targeting Strategy	Photodynamic Approach	Methodology for Assessing the Results	Main Results
[31]	In vitro	Human melanoma cancer A375 cells	CD133 AlPcS_4_Cl-AuNPs	PS: AlPcS_4_Cl Concentration: 35 µM Wavelength: 673.2 nm Light source: low-intensity laser Energy: 75 mW/cm^2^ (5 J/cm^2^) Treatment time: 10.4 s	Flow cytometry Immunofluorescence	Cell viability decreased to 42.7%
[32]	In vitro	Human melanoma cancer A375 cells	CD133 AlPcS_4_Cl-AuNPs	PS: AlPcS_4_Cl Concentration: 35 µM DLI: 4 h Wavelength: 673 nm Light source: low-intensity diode laser Energy: 75 mW/cm^2^ (5 J/cm^2^) Treatment time: 1 min	Microscopy LDH assay ATP assay Trypan blue	Enhanced effect of ROS Decreased cell proliferation
[33]	In vitro	Human Epithelial lung cancer A549 cells	CD133 CD-133 targeted with AuNP-PEG-AlPcS_4_Cl-Ab	PS: AlPcS_4_Cl Concentration: 20 µM DLI: 4 h Wavelength: 673.2 nm Light source: semiconductor diode laser Energy: 10 J/cm^2^	Morphology, LDH cytotoxicity, ATP proliferation, trypan blue viability, and annexin VPI	Localization in the cytoplasm and nuclei of cells due to the anti-CD-133 antibody biomarker
[34]	In vitro	Human breast cancer MCF-7 and MDA-MB-231 Cells	SAL/Ce6@kVE NPs	PS: Ce6 Concentration: 3.38 µM (MCF-7) and 4.95 µM (MDA-MB-231) DLI: 48 h Wavelength: 625 nm Light source: red light Energy: 1 J/cm^2^–20 J/cm^2^	ALDH activity assay Cellular uptake	Higher IC_50_, lower ROS production, and lower activation in apoptotic response Reduced mammosphere formation, enhanced drug penetration
	In vivo			PS: Ce6/SAL Concentration: 1:1:4 ratio DLI: 12 h Wavelength: 625 nm Light source: red light Energy: 20 J/cm^2^	Fluorescence microscopy	
[36]	In vitro	Human esophageal cancer HKESC-1 cells	CD271 CD-271 targeted with AlPcS_4_Cl-AuNPs	PS: AlPcS_4_Cl Wavelength: 673.2 nm Light source: semiconductor diode laser Energy: 5 J/cm^2^	MTT assay, LDH assay and morphology	Less toxicity in non-CSCs, remarkable reduction in viable cells
[37]	In vitro	Human colon carcinoma HeLa cells A375 cells HCT116 cells CD133-positive HCT116 cells	RuL2	PS: Ru complex Concentration: 10 nM–1 μM DLI: 1–5 h Wavelength: 405 and 650 nm Light source: blue and red light Energy: 1–20 mW/cm^2^ Treatment time: 3 min–1 h	MTT assay Cell membrane labeling and imaging. Lipid peroxidation Caspase 3 activity assay Confocal microscopy	Oxidative damage in membrane fatty acids, caspase 3 activation, thus triggering the apoptosis pathway
[38]	In vitro	Human glioblastoma NCH421k cells U251 and NCH241k cells	CD133 AC133 mAb-IR700	PS: IR700 Concentration: 20–40 µg/mL DLI: 6 h Wavelength: 670–710 nm Light source: red LED laser Energy: 12–18 J/cm^2^	Flow cytometry Fluorescence microscopy Western blot	Extension of overall mouse survival induced rapid cell death and shrinkage of both subcutaneous and brain tumors
	In vivo			PS: IR700 Concentration: 100 μg DLI: 24 h Wavelength: 670–710 nm Light source: NIR light Energy: 1–100 J/cm^2^ Treatment time: 14 min and 28 min Treatment duration: two cycles with a 3-day interval	Serum stability assay MRI imaging Histopathology	
[39]	In vitro	Human Colorectal cancer HT-29 and SW620 cells	CD133 CD133–Pyro	PS: Pyro Concentration: 0.25 mg/mL DLI: 2–4 h Wavelength: 670 nm Energy: 5 J/cm^2^	MTT assay Flow cytometry Immunofluorescence Western blot ROS levels	Production of ROS suppresses stemness properties and induces autophagy in CSC Decrease in tumor growth
	In vivo			PS: Pyro Concentration: 2.25 mg/kg DLI: 1.5 h Wavelength: 670 nm Energy: 150 mW/cm^2^ Treatment time: 16 min		
[40]	In vitro	Human Myeloid leukemia 32D-p210-GFP cells Human acute myeloid leukemia cells	CD117 and CD96 CD117-targeted ICG-CPSNPs CD96-targeted ICG-CPSNPs	PS: ICG–CPSNPs Concentration: 10 nM DLI: 1 h–2 h Light source: NIR light Energy: 1 J/cm^2^	Cellular uptake Flow cytometry ROS detection	Decrease in leukemia stem cells’ viability
	In vivo			PS: ICG–CPSNPs Concentration: 200 nM DLI: 30 min Light source: NIR light Energy: 12.5 J/cm^2^ Treatment duration: once every three days		
[41]	In vitro	Human Liver cancer Hepa-1-6 cells Human Breast cancer 4T1 cells	CD133 Ex-apt@LuCXB Ex-apt@LuTex,	PS: LuTex, LuCXB Concentration: 0–40 µM DLI: 24 h Wavelength: 730 nm Light source: NIR laser Energy: 100 W/cm^2^ Treatment time: 10 min	Confocal microscopy CCK-8 Kits γ-H2AX JC-1 detection kit Transwell assay Western blot	Reduced stemness Improved anti-angiogenic effects
	In vivo			PS: LuCXB Concentration: 5 mg/kg (LuCXB) and 0.5 mg/kg (Ex-apt) Wavelength: 730 nm Light source: NIR laser Energy: 0.1 W/cm^2^ Treatment time: 8 min Treatment duration: 2 sessions, 7-day interval, repeated after 7 days	H&E staining Immunohistochemical staining ELISA assay	
[42]	In vitro	Human Breast cancer 4T1 cells	CD44 HCCO	PS: HCCO Concentration: 1.56 µg/mL DLI: 6 h Wavelength: 660 nm Energy: 10 mW/cm^2^ Treatment time: 1 min	Immunofluorescence ROS levels	Inhibits 4T1 cell growth, in vitro and in vivo Effectively inhibits tumor recurrence and metastasis
	In vivo			PS: HCCO Concentration: 3 mg/kg Wavelength: 660 nm Energy: 50 mW/cm^2^ Treatment time: 4 min Treatment duration: 6 times in three-day intervals	H&E and TUNEL staining	
[43]	In vitro	Human Breast cancer MDA-MB-231 cells	CD133 CA9-BPS-Cu(II)	PS: BPS Concentration: 10 µM DLI: 24 h Wavelength: 660 nm Light source: laser Energy: 100 mW/cm^2^ (30 J/cm^2^) Treatment time: 5 min	Aldefluor assay	Decreased stemness triggers apoptosis
	In vivo	Human Breast cancer MDA-MB-231 MDA-MB 453 and T47D cells		PS: BPS Concentration: 800 µM DLI: 1 h Wavelength: 660 nm Light source: laser Energy: 2.0 W/cm^2^ (1200 J/cm^2^) Treatment time: 10 min Treatment duration: once a week for 3 weeks	Fluorescence assay	

**Legend**: ALDH—Aldehyde dehydrogenase; ATP—Adenosine TriPhosphate; DLI—drug-light interval; ELISA—Enzyme-Linked Immunosorbent Assay; IC—inhibitory concentration; JC-1—5,5′,6,6′-tetrachloro-1,1′,3,3′-tetraethylbenzimi-dazolylcarbocyanine iodide); H&E—Haemotoxylin and Eosin; LDH—lactate dehydrogenase; MTT—3-[4,5-dimethylthiazol-2-yl]-2,5 diphenyl tetrazolium bromide; MRI—Magnetic resonance imaging; NIR—Near-Infrared; ROS—Reactive oxygen species; TUNEL—Terminal deoxynucleotidyl transferase dUTP nick-end labeling.

The nanoparticle-based strategies assessed in melanoma, lung, and breast cancer models collectively demonstrate that enhancing photosensitizer delivery and CSC selectivity improves PDT efficacy. CD133-targeted phthalocyanine–gold nanoconjugates consistently increased CSC apoptosis, exhibited strong uptake in CD133^+^ melanoma and lung CSC, and induced apoptotic markers [31,32,33]. In contrast, the multifunctional SAL/Ce6@kVE platform relies on functional CSC selectivity through SAL co-delivery, enabling simultaneous ROS-mediated cytotoxicity and suppression of stemness pathways, including modulation of Wnt/β-catenin signaling in vivo [34]. Despite differences in targeting mechanisms, surface marker specificity versus functional anti-CSC activity, both approaches converge on enhanced ROS generation, reduced CSC viability, and impairment of self-renewal or proliferation. However, the strategies differ in translational potential and technical limitations. The CD133-directed nanoconjugates show strong CSC uptake specificity, but lack reported data on in vivo biodistribution and long-term CSC suppression, which limits assessment of systemic safety and tumor accumulation. Meanwhile, the SAL/Ce6@kVE platform benefits from demonstrated drug loading, pathway modulation, and in vivo efficacy but relies on non-receptor CSC targeting and would require optimization to ensure preferential accumulation in CSC niches.

#### 3.3.2. Ligand-Photosensitizer Conjugates

A PDT approach using the nanoconjugate AlPcS_4_Cl-AuNP-Anti-CD271 was designed to eliminate esophageal CSC. In vitro experiments on HKESC-1 esophageal CSC (CD90^+^/CD44^+^/CD271^+^) showed the accumulation of the nanoconjugate in the nucleus, endoplasmic reticulum, lysosome, and mitochondria. Moreover, nanoconjugate-mediated PDT resulted in significant cytotoxicity and reduced cell viability. Nonetheless, this strategy demonstrated minimal toxicity in normal WS1 fibroblasts [36]. Another in vitro study described three synthesized cyclometalated Ru(II) complexes incorporating substituted benzo[g]quinoxaline ligands and evaluated their photodynamic properties against cervix, melanoma, and colon cancer cells, as well as colon CSC. Experiments were performed on HeLa, A375, and HCT116 cell lines, including CSC-enriched HCT116 populations. Cytotoxicity was assessed, and ROS generation was monitored. Cellular uptake was confirmed, and mechanistic studies evaluated lipid peroxidation and caspase-3 activation. The results demonstrated that light activation significantly enhanced cytotoxicity compared to dark conditions, with blue light producing the most substantial effect. The lead compound localized predominantly in cellular membranes and induced apoptosis through ROS-mediated lipid peroxidation. Importantly, this complex effectively eliminated CSC-enriched populations, with studies in noncancerous MRC5 cells indicating moderate selectivity [37].

A study evaluated a near-infrared (NIR) photoimmunotherapy strategy designed for the selective imaging and eradication of glioblastoma stem cells using a theranostic conjugate (AC133–IR700). In vitro, the conjugate demonstrated high specificity for CD133^+^ glioblastoma stem cells, and PDT treatment induced approximately 80% cell death in CD133-overexpressing U251 cells, whereas minimal cytotoxicity was observed in CD133^−^ cells or cells treated with photosensitizer alone. Morphological alterations consistent with necrotic death were observed post-irradiation. Moreover, patient-derived glioblastoma stem cell spheres retained self-renewal capacity after exposure to irradiation or photosensitizer, whereas PDT treatment nearly abolished sphere formation. In vivo studies were conducted in Harlan immunodeficient nude mice using both subcutaneous (sc) and orthotopic glioblastoma models. Tumor growth was significantly delayed in the irradiated flank compared to the non-irradiated control. In established sc tumors, PDT resulted in significant tumor regression. Treated tumors showed reduced expression of CSC markers (SOX2, Nestin, and Musashi) and increased expression of the differentiation marker GFAP. In the orthotopic model, NCH421k cells were implanted intracranially. Despite the blood–brain barrier, the nanoconjugate accumulated in brain tumors within 24 h of administration. PDT significantly prolonged the survival of mice with orthotopic NCH421k tumors with a single treatment [38]. Another targeting strategy using a novel photosensitizer, CD133–Pyro, was developed by conjugating pyropheophorbide-a to a peptide with high affinity for CD133. In vitro experiments used CSC-enriched (CD133^+^) populations from HT29 and SW620 colorectal cancer cell lines. Confocal microscopy confirmed uptake of CD133–Pyro in HT29 CSC, while CD133^−^ cells showed minimal interaction with CD133–Pyro, demonstrating its selectivity. Further analysis indicated that CD133–Pyro accumulated in the endoplasmic reticulum, lysosomes, and mitochondria of HT29 CSC. PDT with CD133–Pyro significantly reduced sphere formation and self-renewal capacity, and also induced substantial cellular damage in both HT29 and SW620 CSC. For in vivo evaluation, a xenograft model was established by sc injecting CSC-enriched HT29 cells into Balb/c nude mice. Mice treated with PDT presented a significant reduction in tumor growth compared to the control group [39].

To target leukemia stem cells, an NIR-PDT strategy using indocyanine green encapsulated within calcium phosphosilicate nanoparticles was designed. Target specificity was achieved by surface PEGylation and conjugation of anti-CD117 and anti-CD96 antibodies to the nanoparticle surface. In vitro, photodynamic activation of the targeted nanoparticles reduced the CSC-enriched population of a murine leukemia cell line and decreased the viability of two acute myeloid leukemia patient cells. For in vivo evaluation, a leukemia model was established by tail-vein injection of 32D-p210-GFP murine leukemia cells into C3H/HeJ mice. Following nanoparticle administration, localized irradiation was applied to the spleen. This treatment reduced leukemia burden, and 29% of the treated mice achieved disease-free survival. Also, histological examination revealed no significant toxicity in major organs [40].

A recent therapeutic strategy integrates PDT with immunomodulation by developing a lutetium texaphyrin–celecoxib (LuCXB) conjugate. LuCXB functions as a multifunctional small-molecule photosensitizer, combining a cyclooxygenase-2 inhibitory celecoxib unit with a NIR-responsive lutetium texaphyrin scaffold. In aqueous environments, these components undergo intramolecular donor–acceptor interactions, facilitating a unique type-I photodynamic mechanism. Upon exposure to NIR light, LuCXB generates superoxide anions, which induce oxidative stress within tumor microenvironments and promote an immune-activating response. The conjugate design enhances intramolecular electron transfer and increases ROS production even under low-oxygen conditions. To specifically target CSC, LuCXB was encapsulated within engineered exosomes functionalized with a CD133-specific aptamer, resulting in the nanoconstruct Ex-apt@LuCXB. This nanoconjugate exhibited favorable biocompatibility, tumor selectivity, and photosensitivity. In vitro, mouse liver cancer cells (Hepa1-6) treated with Ex-apt@LuCXB-PDT exhibited greater photocytotoxicity and DNA damage than controls. Additionally, this treatment disrupted mitochondrial membrane potential and triggered apoptosis in Hepa1-6 cells. In vivo therapeutic potential was evaluated using a liver cancer model in BALB/c nude mice bearing Hepa1-6 tumors, where intravenous administration of the nanoconstruct led to preferential tumor accumulation, confirmed by fluorescence imaging and colocalization with CD133^+^ cells in tissue sections. In B6 mice, treatment suppressed tumor growth without affecting body weight, and most tumors were eliminated without relapse. Treated tumors showed increased CD4^+^ and CD8^+^ T-cell infiltration, indicating immune activation. The construct was also tested in 4T1 breast tumors, where Ex-apt@LuCXB combined with NIR irradiation produced the most pronounced therapeutic effect, accompanied by reduced angiogenesis, enhanced immune cell infiltration, and cytokine changes, indicating that PDT also stimulates a robust immune response [41].

Hyaluronic acid (HA)-based nanomicelles were engineered to deliver photosensitizers directly to CSC by targeting CD44. These nanomicelles (HCCO) were synthesized using HA as a biocompatible carrier, forming self-assembled structures that encapsulate the hydrophobic photosensitizer Ce6 and Olaparib to inhibit DNA damage repair. In vitro experiments used breast CSC isolated from the 4T1 cell line, characterized by the CD44^+^/CD24^−^ phenotype. PDT significantly suppressed the growth of 4T1 breast CSC. In vivo, Balb/c mice were inoculated with 4T1-CSC to establish an orthotopic model. Animals treated with PDT exhibited greater inhibition of tumor volume. This integrated system induced immunogenic cell death, promoted dendritic cell maturation, and reduced infiltration of myeloid-derived suppressor cells. Additionally, combining PDT with Olaparib resulted in sustained irreversible DNA damage in CSC, supporting the conclusion that this strategy effectively overcame CSC resistance to treatment. PDT also produced substantial inhibition of tumor recurrence and lung metastasis [42].

Another PDT strategy was developed to target CSC that overexpress carbonic anhydrase IX (CAIX), a hypoxia-induced enzyme associated with tumor aggressiveness and therapy resistance. Researchers designed a copper(II)-coordinated BODIPY-based photosensitizer, CA9-BPS-Cu(II), conjugated to a CAIX-targeting ligand. This molecule selectively binds CAIX-expressing cells and induces cytotoxicity upon light activation. Because breast CSC have elevated CAIX, the study investigated CA9-BPS-Cu(II)-based PDT in both in vitro and in vivo breast cancer models. For in vitro analysis, researchers used MDA-MB-231 cells. CD133^+^ cells showed significantly greater sensitivity to conjugate treatment than CD133^−^ cells, likely due to higher CAIX expression. PDT reduced sphere formation, indicating impaired self-renewal and tumor-initiating capacity. Treatment also reduced ALDH1 activity, downregulated key stemness markers such as OCT4 and Nanog, and reduced expression of transcription factors SOX9 and Stat3. For in vivo studies, a xenograft mouse model was established by injecting MDA-MB-231 cells into both femoral regions of Balb/c mice. CAIX expression in tumor tissue was confirmed, as well as a substantial accumulation of the conjugate at the tumor site, with minimal presence in non-target organs. Treatment resulted in a reduction in tumor volume compared with untreated controls and the conjugate group. Similar effects were observed in mice bearing MDA-MB-453 cells, which also express high levels of CAIX. In contrast, mice with T47D tumors (low CAIX expression) showed no significant antitumor effects post-PDT [43].

Across the studies reviewed, CSC-targeted PDT has been achieved using multiple strategies, such as anti-CD271, anti-CD133, anti-CD117/CD96, and CAIX-directed nanoconjugates, which consistently improved photosensitizer localization and enhanced PDT-mediated CSC elimination, as demonstrated in esophageal, melanoma, lung, colorectal, glioblastoma, leukemia, and breast cancer models. Other systems, including Ru(II) complexes, Ex-apt@LuCXB, and HA-based micelles that deliver Ce6 with DNA-repair inhibition, used functional mechanisms, such as ROS-mediated lipid peroxidation, immune activation, inhibition of stemness pathways, or sustained DNA damage. Collectively, these strategies converge in demonstrating potent ROS-induced cytotoxicity, reduced sphere-forming capacity, and downregulation of stemness markers. However, divergence arises from differences in photosensitizer localization (membranes versus endoplasmic reticulum/lysosomes/mitochondria), the degree of CSC reduction, and the robustness of in vivo validation. When comparing translational potential, multifunctional platforms appear most promising, particularly those combining NIR-responsive PDT with immunomodulation (Ex-apt@LuCXB) [41] or receptor-guided delivery to hypoxic CSC niches (CAIX-BPS-Cu(II)) [43]. These systems offer advantages, such as improved biodistribution and sustained suppression of CSC features. Antibody-directed nanoconjugates provide specificity and intracellular accumulation but often lack detailed biodistribution data, which are key for clinical scalability.

### 3.4. Combination Strategies

A growing number of studies have explored the potential of combining PDT with chemotherapy, radiotherapy, or agents that modulate CSC differentiation/inhibition and signaling. The principal findings and design features of these combination systems are summarized in Table 4.

#### 3.4.1. PDT + Chemotherapy

A therapeutic strategy was developed using hyaluronic acid-coated polymeric nanoparticles to co-deliver docetaxel (DTX) and TPCS_2_a. This delivery system specifically targets CD44-overexpressing breast cancer cells, particularly the CD44^+^/CD24^−^ CSC subpopulation. In vitro experiments employed the CD44^high^ MDA-MB-231 and CD24^low^ MCF-7 cell lines. CD44-mediated endocytosis promoted improved nanoparticle cellular uptake, as confirmed in CSC-enriched mammosphere models. Subsequently, PDT resulted in a greater reduction in CSC characteristics, including decreased mammosphere formation and self-renewal capacity, reduced ALDH activity, and a CD44/CD24 phenotype [44].

In recent studies, a hybrid nanoparticle system composed of C60 fullerene and silica, surface-functionalized with hyaluronan, was developed to selectively bind CD44, which is highly expressed in breast CSC. This nanoplatform was designed to co-deliver doxorubicin (DOX) hydrochloride, a chemotherapeutic agent, and indocyanine green. In vitro experiments demonstrated that HA-functionalized nanoparticles efficiently targeted CD44^+^ CSC and delivered the therapeutic agents with high specificity. Mammospheres derived from MCF-7 and MDA-MB-231 human breast cancer cell lines were used as CSC models. The HC60S-DI nanoparticles exhibited significantly enhanced cytotoxicity against mammospheres following NIR laser irradiation. The combined therapeutic effects of the nanoparticles, which integrated chemotherapy, PDT, and photothermal therapy, resulted in effective disruption of breast CSC. In comparison, treatment with DOX or HC60S-DI nanoparticles without irradiation was less effective in reducing cell viability. Mammospheres demonstrate greater resistance to DOX, especially at lower concentrations, than monolayer cell cultures. In vivo studies, we evaluated the therapeutic efficacy and CSC-targeting capability of HC60S-DI nanoparticles in an orthotopic breast cancer model. Female nude mice implanted with MDA-MB-231 mammosphere cells developed tumors enriched in CSC. Mice treated with HC60S-DI nanoparticles with higher drug loading and NIR irradiation exhibited the most pronounced antitumor response, achieving complete tumor ablation in three out of five mice and reduced tumor volumes in the remaining two. Treated tumors showed extensive apoptosis and necrosis and displayed minimal CD44 and CD133 expression, suggesting effective CSC targeting and elimination [45].

A photoactivatable nanoplatform used HA-cholesterol nanoparticles conjugated with pheophorbide A, a photosensitizer for PDT, and SN38, an active metabolite of irinotecan. This approach uses nanoparticles engineered to generate ROS upon light activation of the photosensitizer and to release SN38 through ROS-cleavable thioketal linkages, enabling sequential therapy of PDT and chemotherapy. HA was chosen for its affinity to CD44, facilitating targeted delivery. In vitro studies were performed using HEY-T30 ovarian cancer cells, which overexpress CSC markers (CD44, ALDH1A1, and CD117) and exhibit high invasiveness. These cells were incubated with nanoparticles, which effectively targeted them. After PDT, HEY-T30 treated cells showed cell death rates of 80% and 90%, respectively. In vivo studies using a HEY-T30 xenograft model in BALB/c nude mice showed that nanoparticles preferentially accumulated in tumors and in the liver. Following photodynamic treatment for 3 days, this nanoplatform markedly inhibited tumor growth and induced potent cytotoxicity [46].

A multifunctional nanoplatform was developed to address the limited penetration of nanomedicines into CSC, specifically by employing polydopamine (PDA) nanospheres co-loaded with DOX, gold nanorods, and Ce6 to create the PDA@GNRs-DOX/Ce6 system. After systemic administration, this nanosystem accumulated in tumor tissues due to the enhanced permeability and retention (EPR) effect. Upon NIR laser irradiation, the nanostructure disassembled, enabling localized DOX release and singlet oxygen generation by Ce6, effectively destroying superficial tumor cells. Simultaneously, smaller nanorods detached and penetrated deeper into the tumor mass, facilitating targeted destruction of CSC in hypoxic niches. In vitro, the combination of photothermal therapy with chemotherapy using the system significantly reduced cancer cell viability, leaving approximately 20% viable HeLa cells; further dual-laser treatment enhanced efficacy, reducing survival to about 15%. Almost all irradiated cells were eradicated under dual-laser exposure. In vivo biodistribution studies demonstrated substantial tumor accumulation of the system and subsequent clearance of the gold element after seven days. In efficacy tests, U14 cell-injected Kunming mice received intravenous nanoformulations, followed by targeted laser irradiation. Mice from the saline and system groups were also irradiated. The system-treated group exhibited a 26-fold lower tumor growth rate compared to controls and induced tumor cell apoptosis [47].

According to studies, HA-based delivery systems emerged as a recurrent strategy for enhancing PDT selectivity toward CSC, largely through CD44-mediated uptake. HA-coated nanoparticles co-delivering chemotherapeutics and photosensitizers (DTX/TPCS_2_a, DOX/indocyanine green, and SN38/PheoA) [46,47,52] consistently improved nanoparticle internalization in CD44^+^ breast and ovarian CSC, yielding reductions in mammosphere formation, stemness markers, and ALDH activity. Multifunctional platforms that integrate chemotherapy, PDT, and photothermal therapy (such as HC60S-DI) [45] produced especially potent CSC eradication, with in vivo studies reporting complete tumor ablation in some cases. Meanwhile, systems incorporating ROS-responsive photosensitizers and sequential release mechanisms, such as HC-NP-PheoA-SN38 [46], effectively combined PDT with controlled chemotherapeutic delivery. A complementary design, PDA@GNRs-DOX/Ce6 847], addressed poor CSC penetration by enabling deeper intratumoral diffusion of gold nanorods upon NIR activation, facilitating CSC elimination even in hypoxic niches. Together, these approaches converge on the principle that nanoparticle engineering enhances the potency of PDT and CSC suppression. Despite these similarities, distinctions influence overall translational potential. HA-based targeting provides robust CSC specificity but relies on CD44 expression, which may vary across tumors. Nanoplatforms offer improved multifunctionality but can face challenges related to formulation complexity and potential off-target biodistribution. Although several studies demonstrated favorable tumor accumulation, biodistribution patterns differed. The most promising strategies appear to be those combining targeted uptake with multi-modal mechanisms, chemotherapy, PDT, and photothermal therapy, as these platforms consistently show superior CSC elimination, reduced tumor recurrence, and more robust in vivo validation.

#### 3.4.2. PDT + Radiotherapy

A biomimetic mesoporous organosilicon nanosystem, referred to as PMT, was designed to precisely eliminate CSC and prevent tumor recurrence following radiotherapy. The PMT system consisted of mesoporous organosilicon nanoparticles loaded with a type-I aggregation-induced emission photosensitizer (TBP-2) and coated with a platelet membrane to enhance tumor targeting and immune evasion. The nanosystem was engineered to degrade in response to intracellular glutathione, releasing TBP-2 for PDT. Upon exposure to white light, TBP-2 generated hydroxyl radicals through a type-I PDT mechanism, promoting CSC death even under hypoxic conditions. Breast CSC were obtained as spheres from the 4T1 cell line and were treated with PMT. After treatment, spheroid formation was almost completely suppressed in the PMT-irradiation group, with a formation rate of less than 5%. Cell viability assays confirmed that PMT reduced CSC viability in a dose-dependent manner. Subsequent experiments assessed the combined antitumor effects of PMT-mediated PDT and radiotherapy. This combination significantly increased DNA damage in CSC populations. In vivo studies used a 4T1 breast cancer heterotopic model in Balb/c mice to assess biodistribution and pharmacokinetics, as well as therapeutic effect. A pharmacokinetic analysis revealed that PMT exhibited a markedly prolonged circulation time. Further investigations confirmed that PMT demonstrated strong tumor-targeting capability, with substantial accumulation around cells expressing high levels of CD44. To evaluate therapeutic efficacy, mice were treated with PMT, irradiated for 2 consecutive days, followed by radiotherapy. After treatment, tumor volumes decreased rapidly, and no recurrence was observed at 30 days. Mice receiving PMT, combined with light and radiotherapy, also exhibited prolonged survival. Histological analyses, including TUNEL, Ki-67, and H&E staining, revealed more extensive tumor cell damage in this group. Additionally, ALDH1 expression was markedly reduced. Importantly, no significant weight loss or systemic toxicity was detected, indicating that PMT possesses favorable biocompatibility and safety [48].

A similar aggregation-induced emission-based nanotherapeutic system was designed to enhance the efficacy of FLASH radiotherapy, prevent tumor recurrence, and minimize side effects in colon cancer. This system comprises TPT nanoparticles coated with platelet-derived membranes, which extend their circulation time and facilitate their accumulation in hypoxic tumor regions, where CSC are commonly found. The inclusion of tantalum increased the local radiation dose due to its high atomic number. Additionally, TBP-2 showed strong photodynamic effects under white light, thereby boosting X-ray-induced cytotoxicity by generating ROS even under low-oxygen conditions. Colon CSC were isolated from CT26 cells using spheres with the CD44^+^/CD133^+^ phenotype. Nanoparticles exhibited minimal cytotoxicity and good biocompatibility when incubated with CT26 cells, CSC, or normal cells. Under hypoxic conditions, cell viability decreased to 81.3% following FLASH radiotherapy and further dropped to 60.3% when combined with TPT treatment. In comparison, only 35.7% of cells survived after combined treatment with TPT, light, and FLASH. This last combination significantly reduced the sphere-forming ability of CSC, resulting in a lower number of spheres, and nearly all tumor cell colonies were eradicated. By subcutaneously inoculating CT26 CSC into Balb/c mice, a heterotopic model was established to evaluate biodistribution, pharmacokinetics, and therapeutic potential. Thirty days after TPT nanoparticle administration, no signs of organ damage were observed, confirming the nanoparticles’ biosafety. Biodistribution studies revealed that nanoparticles predominantly accumulated in the liver. Pharmacokinetic analysis showed that TPT nanoparticles exhibited prolonged circulation, reducing rapid clearance. After TPT + light and FLASH, tumor growth suppression was observed during the first 10 days post-treatment [49].

Across biomimetic and aggregation-induced emission-based PDT strategies, a common theme is the integration of tumor-homing nanosystems with ROS-generating photosensitizers, particularly in hypoxic or radioresistant niches. Both PMT and TPT platforms used platelet-membrane coatings to prolong circulation and enhance accumulation in CSC-rich CD44^high^ regions, while glutathione-responsive release of TBP-2 supported potent type-I PDT even under low oxygen. These systems consistently reduced CSC viability, sphere formation, and stemness markers and showed synergy with radiotherapy or FLASH irradiation [48,49]. However, differences emerged. PMT demonstrated tumor-specific localization and more robust radiosensitization in breast CSC, whereas TPT showed effective CSC elimination but accumulated more heavily in the liver, indicating biodistribution limitations. Overall, multifunctional platforms combining biomimetic targeting, degradable carriers, and PDT–radiotherapy synergy appear the most promising for durable CSC eradication, although further optimization is needed to address organ accumulation and light-penetration limitations.

#### 3.4.3. PDT + CSC Differentiation Agents or Inhibitors

A self-assembled nano-prodrug system was created, integrating an ALDH-activatable photosensitizer with disulfide-linked all-trans retinoic acid (ATRA). In the presence of high ALDH levels, the nanoformulation selectively disassembles in the high-glutathione environment of CSC, releasing ATRA and the photosensitizer. In the breast 4T1 CSC-enriched microenvironment, ALDH activates the photosensitizer (PS-CHO@ATRA-SS-ATRA). This occurs through the conversion of aldehydes into carboxylic acid moieties, which triggers the production of ROS and a strong fluorescence signal. In turn, this enables both therapeutic action and imaging. ROS directly eliminate CSC, and releasing ATRA promotes differentiation and reduces stemness markers (Nanog, SOX2, and OCT4), thereby enhancing therapeutic efficacy. In vivo studies using a breast heterotopic mouse model in Balb/c mice demonstrated accumulation of the nanoformulation in CSC-rich regions. PDT significantly inhibited tumor growth. In an orthotopic breast cancer model established with 4T1 cells in Balb/c mice, photodynamic treatment with these agents suppressed tumor growth and reduced lung and liver metastasis [21].

A carrier-free nanodrug system was developed to enhance the efficacy of PDT and simultaneously inhibit stemness. MKCe6 nanoparticles were self-assembled from two functional molecules, Ce6 and MK-0752, a γ-secretase inhibitor that blocks the Notch signaling pathway, which is critical for CSC maintenance and therapy resistance. In vitro studies used HCT116 human colon cancer cells, including CSC-enriched populations. PDT was applied using NIR laser irradiation, although the exact irradiation time and other relevant parameters were not specified. To assess sphere-forming capacity, CD24^+^/CD44^+^ HCT116 cells were sorted. CSC treated with nanoparticles showed reduced sphere formation, likely due to a decrease in the CSC proportion. Additionally, HCT166 CSC treated with nanoparticles were more sensitive to PDT than HCT166 cells. Nanoparticles also suppressed cell migration and invasion. In vivo, Balb/c nude mice inoculated with HCT166 cells received systemic nanoparticle administration, resulting in efficient tumor accumulation. Tumor growth was inhibited, and expression of stemness markers (CD133, OCT4, and SOX2) in tumor tissues decreased [50].

Another multifunctional nanoplatform HP@PP/LC, was developed to enhance PDT against breast CSC by simultaneously promoting their differentiation and inhibiting EMT, a process that contributes to CSC regeneration and metastasis. The nanoplatform was constructed by encapsulating haloperidol, a dopamine receptor antagonist known to induce CSC differentiation, within polyethyleneimine–polyhistidine micelles. These micelles were then conjugated to a low-molecular-weight heparin–Ce6 conjugate to form HP@PP/LC nanoparticles. The micelle component becomes protonated in the acidic tumor microenvironment, thereby facilitating the targeted delivery of haloperidol to breast CSC. Low-molecular-weight heparin blocks EMT signaling. In vitro tests were performed using the human breast cancer cell line 4T1, which was enriched for CSC through a mammospheres protocol. Breast CSC were characterized regarding the CD44^+^/CD24^−^ phenotype, and cell death-associated PDT was evaluated. The results demonstrated that nanoparticles effectively promoted the differentiation of breast CSC into non-stem tumor cells, thereby reducing their self-renewal capacity. Upon laser activation at pH 6.5, nanoparticles induce enhanced apoptosis and necrosis in 51.53% of breast CSC. Additionally, EMT was successfully inhibited by increasing E-cadherin expression and decreasing N-cadherin upon treatment with nanoparticles. Mice bearing orthotopic breast cancer xenografts received nanoparticles followed by laser irradiation at the tumor site. Evaluations were performed at days 1, 3, 7, and 14 post-treatment. Tumor growth was markedly inhibited, and EMT was blocked (upregulation of E-cadherin and downregulation of N-cadherin). Moreover, the proportion of CD44^+^/CD24^−^ cells decreased in animals treated with nanoparticles compared with the other groups [51].

To induce CSC differentiation, a multifunctional nanotherapeutic platform, SEED nanoparticles, was developed. This platform enables targeted delivery with site-specific drug release that responds to the tumor microenvironment. SEED nanoparticles incorporate three components, DOX, Ce6, and ATRA, to induce CSC differentiation. The design allows a dual-response mechanism. In non-CSC regions, ROS generated by PDT rapidly releases DOX and Ce6, thereby exerting a combined chemo-PDT effect. In CSC niches, ATRA is preferentially released to promote differentiation, making CSC more susceptible to chemotherapy. In vitro, 4T1 breast cancer and CSC-enriched (CD44^+^/CD24^−^) cells were used. SEED nanoparticles were incubated with cells for 24 h, followed by laser irradiation. SEED nanoparticles led to greater downregulation of stemness markers (CD44 and SOX2), increased ROS levels, and enhanced cytotoxicity compared with single-modality treatments. This ROS-driven effect supports further CSC differentiation and drug release, creating a positive feedback loop that boosts treatment efficacy. For in vivo therapeutic evaluation, CSC-enriched 4T1 orthotopic tumors were established in Balb/c mice. Treatment showed that SEED nanoparticles could eliminate tumors without apparent systemic toxicity [52]. Another therapeutic approach that combines differentiation therapy with PDT to selectively target and eliminate breast CSC and their progeny was created. A biomimetic nanoplatform co-loaded with the NIR photosensitizer IR-780 and the differentiation agent ATRA was engineered and assembled via a protein-guided biomineralization process that incorporated aluminum ions. These nanoclusters were engineered to degrade under acidic conditions, facilitating deep tumor penetration and controlled drug release. ATRA drives CSC differentiation, reducing their stem-like properties and making them more susceptible to treatment. In vitro studies were performed using mammospheres from the 4T1 breast cancer cell line. After CSC characterization of the stemness profile (CD44^+^/CD24^−^), spheres were treated with the nano-preparations IR/AT@HPOC and M1-IR/AT@HPOC. When the combined impact of ATRA and IR-780 on CSC elimination in vitro was assessed, both agents resulted in markedly increased cytotoxicity against CSC populations. Experimental observations indicated that approximately 33.6% of CD44^+^/CD24^−^ cells were present in mammospheres, whereas their occurrence in untreated 4T1 cells was less than 3%. While IR-780 alone modestly reduced the CD44^+^/CD24^−^ ratio, the addition of ATRA significantly diminished mammosphere stemness, lowering the percentage from 37.1% to 23.0%. The PDT treatment group showed the greatest effect, reducing the CD44^+^/CD24^−^ population from 37.1% to 12.8%. Additional analyses confirmed that treated spheres exhibited substantial downregulation of OCT4 and SOX2 expression, along with minimal colony formation. Moreover, treatment significantly reduced both the number and size of mammospheres. In vivo studies employed 4T1 breast tumor-bearing Balb/c heterotopic mouse models to assess therapeutic efficacy and to investigate the effect of PDT on breast metastasis. The therapeutic strategy demonstrated the ability to inhibit both primary and distant tumor growth, as well as pulmonary metastasis. Moreover, it induced tumor-specific immune memory, providing long-term protection against cancer recurrence upon rechallenge in mice [53].

A dual-acting nanotherapeutic platform was developed, combining a hemicyanine-based photosensitizer (CyOA) for PDT with a glutaminolysis inhibitor (HYL001) to enhance oxidative stress and eliminate CSC. The nanoparticles were assembled using folic acid–hydroxyethyl starch conjugates for tumor-specific delivery. Mechanistically, PDT generates ROS, while glutaminolysis inhibition reduces intracellular antioxidants (GSH and NADPH) and limits oxidative phosphorylation, thereby conserving oxygen for PDT and amplifying ROS-mediated cytotoxicity. In vitro, breast CSC derived from MDA-MB-231 cells were treated with nanoparticles and irradiated. ROS generation was quantified using DCFH-DA probes, and cell viability was assessed. Western blot and flow cytometry confirmed significant downregulation of CSC markers (ALDH1 and SOX2) and increased apoptosis compared with PDT alone. Glutaminolysis inhibition was verified by reduced GSH and NADPH levels, indicating effective metabolic disruption. In vivo, BALB/c nude mice bearing 4T1 orthotopic xenografts enriched with CSC received intravenous injections of nanoparticles followed by localized laser irradiation. A prominent reduction in tumor growth was observed, and histological analyses revealed extensive apoptosis and necrosis in treated tumors. Immunohistochemistry confirmed suppression of CSC markers. No significant toxicity was observed in major organs [54].

A multifunctional nanoplatform combining vascular-normalization therapy with light-activated treatments to target breast CSC was developed. The system consisted of an acid-sensitive conjugate (F3 peptide–heparin–gambogic acid) designed to normalize tumor vasculature, with zinc phthalocyanine (ZnPc) encapsulated to enable photodynamic and photothermal therapy. In the first treatment phase, mild PDT (671 nm) generated moderate ROS levels that promoted CSC differentiation and supported vascular normalization. The second phase used photothermal therapy (808 nm) for tumor ablation. Breast CSC, identified as CD44^+^/CD24^−^ cells from 4T1 spheres, efficiently internalized the nanoplatform via F3-mediated uptake. In vitro, FLG/ZnPc-based photothermal therapy reduced sphere number and integrity in a concentration-dependent manner. In an orthotopic 4T1 CSC model in Balb/c mice, groups receiving photothermal therapy, particularly the combined regimen, showed markedly enhanced tumor suppression. After day 16, a triple-modality treatment (FLG/ZnPc + PDT + photothermal therapy) achieved superior and sustained tumor inhibition, with some mice experiencing complete tumor regression. This combination achieved a tumor growth inhibition rate of ~97% and produced the lowest Ki-67 expression, indicating reduced tumor cell proliferation [55].

Converging evidence shows robust reductions in sphere formation/self-renewal, downregulation of stemness markers (Nanog, SOX2, OCT4, ALDH1, and CD44/CD24), suppressed migration/invasion, and significant in vivo tumor control. Systems accumulated effectively in tumors and CSC-rich niches, with favorable biosafety.

#### 3.4.4. PDT + Chemotherapy + CSC Inhibitors

A biomimetic nanobomb system was designed to achieve synergistic cancer therapy by combining PDT, chemotherapy, and CSC inhibition. The nanoplatform consisted of a core loaded with PDA, a photosensitizer, and chemotherapeutic agents, coated with a cancer cell membrane to enhance tumor targeting and immune evasion. The system was engineered to release its payload in response to intracellular stimuli, such as acidic pH and ROS, ensuring controlled drug delivery within tumor cells. The findings indicated that treatment with MnOx/PDA effectively suppressed the spheroid-forming capacity of colorectal cancer HT29 and RKO cells, suggesting reduced self-renewal potential. Both the number and size of primary and secondary tumor spheres were markedly decreased following MnOx/PDA combined with laser therapy, providing strong evidence that this synergistic approach impairs the self-renewal properties of CSC. Additionally, ALDH1A1, OCT4, and SOX2 expression were downregulated in colorectal cells following treatment. Also, the Western blot analysis of EMT-related markers in HT29 and RKO cell lines revealed that MnOx/PDA treatment led to an increase in E-cadherin levels, while Snail, Slug, and vimentin levels dropped significantly by 56%, 85%, and 52% in HT29 cells and by 89%, 92%, and 80% in RKO cells, respectively. Following the establishment of a colorectal cancer model, animals were subjected to intravenous administration of MnOx/PDA nanobombs, and accumulation within tumor tissue was exhibited. Additionally, no significant toxicity was detected, suggesting favorable biocompatibility. MnOx/PDA@M-based treatment triggered extensive apoptosis and necrosis in cancer cells. A histological analysis of major organs revealed no evident pathological changes, further supporting the safety profile of this therapeutic approach. Additionally, key stemness-associated markers, including ALDH1A1, Snail, and SOX2, were downregulated [56].

The study presents encouraging results, but several limitations must be acknowledged. Most experiments were conducted in vitro and in a subcutaneous tumor model, which does not fully represent the behavior of CSC in their native microenvironment. Although the cancer cell–membrane coating improved tumor targeting, its biological variability and limited scalability may restrict clinical translation. The study also lacked detailed biodistribution and pharmacokinetic data, making it difficult to assess long-term accumulation or potential off-target accumulation.

### 3.5. Photochemical Internalization

Photochemical internalization (PCI) is a widely adopted approach for targeting surface markers on CSC. This method enhances the intracellular delivery of macromolecules from endocytic compartments to the cytosol. PCI utilizes photosensitizing agents that are sequestered within endocytic vesicles. When exposed to specific wavelengths of light, these agents induce vesicle disruption and promote the release of their contents into the cytoplasm. PCI has been shown to increase the cellular uptake and therapeutic efficacy of various macromolecules and compounds that otherwise exhibit limited membrane permeability [57,58]. The studies using PCI as a strategy are summarized in Table 5.

PCI has been utilized to enhance the cytotoxicity of an immunotoxin targeting CD133-expressing CSC-derived cells in colon cancer. The immunotoxin comprises the CD133/1 monoclonal antibody conjugated to saporin (anti-CD133/1-sap). Fluorescence microscopy demonstrated specific antibody binding and uptake, while confocal imaging confirmed colocalization with the photosensitizer TPCS_2_a in endosomal and lysosomal compartments. When conjugated to TPCS_2_a, anti-CD133/1-sap exhibited potent and highly selective toxicity at femtomolar concentrations. CD133^high^ WiDr cells displayed increased sphere-forming capacity and greater tumorigenic potential in vivo, as validated in athymic nude Foxn1nu mice, compared to CD133^low^ cells, which were more sensitive to PDT than CD133^high^ and parental lines. However, following anti-CD133/1-sap plus TPCS_2_a treatment, PCI in CD133^low^ cells was six times higher than with PDT alone and 28 times higher in CD133^high^ cells [59]. Although these results are promising, this strategy relies exclusively on CD133 as a CSC marker. Given the heterogeneity of CSC populations, this reliance may limit applicability across tumor types. A comparable methodology was applied to SW872 and HT-1080 sarcoma cells, both of which contained a small but distinct CD133^high^ subpopulation, to evaluate the efficacy of PCI in delivering CD133-targeted immunotoxins. These immunotoxins were generated by conjugating the ribosome-inactivating protein saporin to two monoclonal antibodies, CD133/1 (AC133) and CD133/2 (293C), each targeting specific CD133 epitopes. Light-activated PCI using TPCS_2_a resulted in significant cytotoxicity in both SW872 and HT1080 sarcoma cells expressing CD133. To assess the tumorigenic potential of CSC, the ability to form three-dimensional colonies and generate tumors in vivo (NOD-scid IL2Rgammannull) was evaluated. Cells that survived PCI treatment demonstrated a marked reduction in proliferation, resulting in decreased colony formation and lower tumorigenic potential [60]. The CD133-targeting immunotoxin AC133–saporin was also evaluated in colorectal cancer, breast cancer, and melanoma. In CD133^high^ colorectal cancer cells (WiDr), PCI with picomolar concentrations of AC133–saporin completely inhibited viability and colony formation, with no toxicity observed in the absence of light activation. Similar efficacy was observed in CD133^+^ breast (MDa-MB-231) and CD133^high^ melanoma cells (FMEX-1) but not in CD133^−^ breast cancer cells (MCF7), confirming target specificity. Mechanistically, PCI-AC133–saporin induced necrosis, S-phase arrest, and disrupted autophagic flux. In vivo, PCI was tested in athymic nude Foxn1nu mice subcutaneously inoculated with WiDr cells treated with AC133–saporin and TPCS_2_a. Administration of a single systemic dose followed by PCI resulted in significant in vivo antitumor effects [61]. In addition to the limitations already mentioned for the studies described earlier in this section, the in vivo model employed in this study assessed only short-term antitumor response rather than long-term outcomes, such as recurrence or metastasis. Later, an engineered construct, scFvCD133/rGelonin, was developed by fusing a single-chain variable fragment (scFv) that recognizes both glycosylated and non-glycosylated forms of human and murine CD133 with the ribosome-inactivating protein gelonin. This construct was evaluated as a novel immunotoxin targeting CD133 and designed for enhanced intracellular delivery through PCI. The experimental design included the CD133^high^ (WiDr and HT29), CD133^low^ (MDA-MB-231 and U87), and CD133^−^ (MCF7 and NIH/3T3) cell lines, with cytotoxicity measured using the MTT assay. scFvCD133/rGelonin demonstrated strong affinity for CD133^high^ cells and accumulated more efficiently in these cells than in CD133^low^ cells, but it exhibited minimal cytotoxicity alone, even at high concentrations. In contrast, when combined with PCI, the immunotoxin induced a pronounced dose-dependent reduction in cell viability [62]. This study was limited to in vitro experiments. Therefore, detailed analysis of the mechanism of action or in vivo validation may represent a relevant future prospect for subsequent clinical translation. A similar strategy was employed to target CD44 in seven distinct human cancer cell lines, including colorectal (WiDr), breast (MDA-MB-231), pancreatic (BxPC-3 and MIA PaCa-2), prostate (DU 145 and LNCaP), and liposarcoma (SW872) cells. The immunotoxin comprised the pan-CD44 monoclonal antibody IM7 conjugated to the ribosome-inactivating protein saporin using a biotin–streptavidin bridge (IM7-saporin). Epifluorescence microscopy demonstrated CD44-specific binding and internalization of the fluorescently labeled antibody (IM7-Alexa488), as well as its colocalization with the PCI photosensitizer TPCS_2_a. Upon light activation, PCI treatment induced effective and selective cytotoxicity in CD44^+^ cells, whereas CD44^−^ cells exhibited minimal response. In addition, CD44^+^ prostate cancer cells were resistant to PDT, potentially due to lower ROS levels than those in CD44^−^ cells [63]. Consistent with previous research, this study was limited to in vitro experiments and lacked in vivo validation. Furthermore, the exclusive focus on CD44 as a marker cannot account for the heterogeneity of CSC populations.

**Table 5:** Studies addressing photochemical internalization.

Ref.	Model	Disease/Source of CSCs	Targeting Strategy	Photodynamic Approach	Methodology for Assessing the Results	Main Results
[62]	In vitro	Human colon adenocarcinoma WiDr and Ht29 cells Human breast cancer MDA-MB-231 and MCF7 cells Human Murine fibroblastoma NIH/3T3 cells Human glioblastoma U87 cells	scFvCD133/rGelonin	PS: TPCS2a Concentration: 0.4 mg/mL DLI: 18 h Wavelength: 570 nm Light source: blue light Energy: 13.5 m W/cm^2^	Western blot assay Flow cytometry Live-cell fluorescence microscopy	Induced log-fold reduction in viability PCI promotes cytotoxic activity Sensitivity is not dependent on CD133 expression by cells
[63]	In vitro	Human Breast carcinoma MDA-MB-231 cells Human Colorectal cancer WiDr cells Human Pancreatic cancer BxPC-3 and MIA PaCa-2 cells Human Prostate cancer DU 145 and LNCaP cells	CD44-targeted IM7-Saporin	PS: AlPcS_2_a and TPCS2a Concentration: TPCs2a (0.35 µg/mL) and AIPcs2a (5 mg/mL) DLI: 18 h Wavelength: 620 nm Light source: red light Energy: 1.5 mW/cm^2^ and 12 mW/cm^2^ Treatment time: 0 to 20 min	Fluorescence microscopy Flow cytometry ROS detection	Strong cytotoxic response CD44-expressing cell lines were highly sensitive to treatment
[59]	In vitro	Human Colorectal cancer WiDr cells	TPCS_2a_-PCI of anti-CD133/1-sap	PS: TPCS_2a_ Concentration: 0.8 pM DLI: 18 h Wavelength: 435 nm Light source: blue light Energy: 11.5 mW/cm^2^ (2.07 J/cm) Treatment time: 3 min	Immunohistology Immunocytology	Highly selective for tumors
	In vivo					
[60]	In vitro	Undifferentiated human sarcoma HT-1080 SW872 cells	TPCS_2a_/AC133–saporin	PS: TPCS2a Concentration: 0.4 mg/mL DLI: 4–18 h Wavelength: 435 nm Light source: blue light Energy: 11 mW/cm^2^ Treatment time: 0–180 s	Confocal microscopy MTT assay Colony-forming assay Tumor-forming assay CSC assays	Low cell viability Reduced colony formation capacity and tumorigenicity
	In vivo			PS: TPCS2a Concentration: 0.8 mg/mL DLI: 4 h Wavelength: 435 nm Light source: blue light Energy: 11 mW/cm^2^ Treatment time: 4 min		
[61]	In vitro	Breast cancer MDa-MB-231 Melanoma FMEX-1 Colorectal cancer WiDr cells	CD133-targeted AC133-mAb-saporin with TPCS2a	PS: TPCS2a Concentration: 0.2 µg/mL DLI: 4 h Wavelength: 652 nm Light source: red diode laser Energy: 90 mW/cm^2^ (15 J/cm^2^) Treatment time: 60 to 180 s	MTT assay Fluorescence microscopy Western immunoblot	Decreased viability and colony formation Growth delay and activated caspase 3 activity
	In vivo	Colorectal cancer WiDr cells		PS: TPCS2a Concentration: 5 mg/Kg DLI: 72 h Wavelength: 652 nm Light source: red diode laser Energy: 90 mW/cm^2^ (15 J/cm^2^) Treatment time: 60 to 180 s	H&E staining Immunohistochemistry	

**Legend**: CSC—Cancer stem cells; DLI—drug-light interval; H&E—Haemotoxylin and Eosin; PCI—photochemical internalization; MTT—3-[4,5-dimethylthiazol-2-yl]-2,5 diphenyl tetrazolium bromide; ROS—Reactive oxygen species.

In this section, all the studies were limited to in vitro experiments, which significantly restricts the translational relevance of these findings. From the malignant diseases addressed, colorectal cancer was the most frequently studied tumor type, followed by breast cancer. This focus reflects the recognized role of CSC in these malignancies but does not capture the full heterogeneity of CSC-driven tumors. Interestingly, all studies used the same photosensitizer, TPCS_2_a, which is commonly used in PCI strategies due to its ability to facilitate endosomal escape of therapeutic agents. While this consistency simplifies comparisons, it limits insights into alternative photosensitizers that might offer improved tissue penetration or pharmacokinetics.

PCI-based approaches also present technical challenges as they require precise light delivery, which is feasible for superficial tumors but can be difficult for deep tumors. Overall, the current evidence suggests that, while PDT combined with CSC-targeting strategies holds promise, significant challenges remain regarding marker selection, safety, technical feasibility, and long-term efficacy before clinical translation can be considered.

## 4. Main Considerations

This scoping review demonstrates increasing interest in PDT as a targeted strategy for eradicating CSC. The elimination of CSC remains a significant challenge in oncology due to their unique biological characteristics and intrinsic resistance mechanisms, which allow them to evade conventional therapies and repopulate tumors [64].

One relevant consideration emerging from the PDT literature is the substantial heterogeneity in how CSC are identified, reflecting broader conceptual variability. Although CSC represent a biologically distinct tumor subpopulation, they are operationally defined using different criteria across studies, including surface-marker expression, enzymatic activity, and functional assays. This inconsistency has direct implications for how CSC-targeted PDT is evaluated and compared. In the studies included in this review, several relied on surface markers, where the specificity, stability, and context dependence of these markers vary widely across cancer types and even across cell lines, limiting their reliability as universal CSC identifiers. Other studies defined CSC through ALDH enzymatic activity, the presence of a side-population phenotype, or through functional assays, such as sphere-forming capacity, each of which captures different aspects of stemness. While these approaches are well-established, the lack of a unified CSC definition contributes to methodological variability and can complicate the interpretation of PDT outcomes across experimental studies.

In this context, while three-dimensional sphere cultures provide a more physiologically relevant model for studying CSC behavior, conventional two-dimensional models can face limitations by exposing all cells uniformly to nutrients and oxygen. Spheres naturally generate outer proliferative zones and inner hypoxic or even necrotic cores that can recapitulate CSC niches in vivo and better mirror the complexity of the tumor microenvironment.

Hypoxia is a well-established characteristic of many CSC niches, arising from the abnormal and inefficient vasculature found in solid tumors [65]. CSC frequently localize to these poorly oxygenated regions, where low oxygen tension contributes to the maintenance of stemness, metabolic adaptation, and resistance to oxidative stress [66]. This microenvironmental feature has direct implications for PDT, particularly for photosensitizers that rely on type II photochemical reactions, where oxygen is an essential substrate for singlet oxygen generation. Under reduced-oxygen conditions, singlet oxygen production is diminished, limiting cytotoxicity. Additionally, the hypoxic microenvironment may simultaneously upregulate antioxidant defenses that counteract radical ROS [67], creating heterogeneous and context-dependent PDT responses.

Despite the biological relevance of hypoxia, many PDT studies included in this review were carried out under normoxic in vitro conditions, which do not accurately replicate CSC microenvironments. Normoxic cultures may enhance PDT efficacy by ensuring adequate oxygen availability and reducing the activation of hypoxia-driven survival pathways. As a result, comparisons between studies performed under normoxia and those incorporating hypoxic conditions must be interpreted with caution. The efficacy reported in normoxic studies may not translate to the physiological niches where CSC reside during tumor progression.

Despite these barriers, several PDT strategies can overcome CSC defenses under appropriate conditions. PDT that favors type I photochemical pathways, which generate radical species (superoxide anion, hydrogen peroxide, and hydroxyl radicals), may be less dependent on oxygen availability [68] and may penetrate more effectively in hypoxic CSC niches. Targeting specific organelles, particularly mitochondria and lysosomes, can enhance PDT efficacy against CSC by disrupting metabolic homeostasis or triggering cell death pathways [69]. Strategies that improve oxygen availability or increase intracellular ROS upon irradiation also show promise in sensitizing CSC to PDT. PDT modalities that operate effectively under hypoxic conditions, generate radical species through type I reactions, or exploit organelle-specific vulnerabilities offer promising paths for overcoming these CSC-associated defenses.

Although no clinical studies have yet evaluated CSC-targeted PDT, several translation barriers must be considered. Beyond the previously mentioned technical and biological considerations, other limitations can also constrain the clinical applicability of CSC-targeted PDT. CSC plasticity represents an inherent biological challenge: even if PDT successfully eliminates existing CSC, non-stem tumor cells may reacquire stem-like properties under stress [5]. This means that therapies aimed exclusively at eliminating current CSC populations may fail to prevent the re-emergence of stem-like cell states after treatment. Additionally, because no single marker universally defines CSC across tumor types, approaches that rely on highly specific targeting may have limited translational robustness, further highlighting the need for strategies that combine multiple markers, exploit functional phenotypes, or modulate the tumor microenvironment to overcome CSC permanence. A structured understanding of these challenges is essential for guiding future research and for identifying PDT modalities with the greatest potential for clinical relevance.

## 5. Conclusions

Over the last few years, many PDT-based strategies have been developed and tested both in vitro and in vivo to eliminate CSC. In general, most strategies developed converge on demonstrating reduced sphere formation, downregulation of stemness markers, impaired self-renewal, and potential in vivo tumor suppression. However, progress is limited by some technical and biological considerations, such as substantial heterogeneity in CSC definitions and isolation methods, and a lack of more robust validation studies. Overall, the most promising approaches appear to be multifunctional platforms that allow selective PDT to disable CSC maintenance pathways or promote differentiation. Nevertheless, the clinical application of these potential strategies requires further investigation via more standardized methodologies and comprehensive translational assessment.

## Figures and Tables

**Table 2:** Studies addressing microenvironment-responsive PDT systems.

Ref.	Model	Disease/Source of CSC	Targeting Strategy	Photodynamic Approach	Methodology for Assessing the Results	Main Results
[29]	In vitro	Human Breast cancer 4T1 cells	NPs	PS: m-TAPP Concentration: 0–100 mg/mL DLI: 24 h Wavelength: 660 nm Energy: 1.25 W/cm^2^ Treatment time: 5 min	MTT assay Flow cytometry	Achieved ribosome degradation and induced apoptosis in cancer cells Metastatic potential reduction
	In vivo			PS: m-TAPP Concentration: 200 mg/kg DLI: 24 h Wavelength: 660 nm Energy: 1.25 W/cm^2^ Treatment time: 10 min	H&E staining TUNEL staining Immunofluorescence assay	
[30]	In vitro	Human Breast cancer MDA-MB-231 cells	PGDAT@N	PS: m-TAPP Concentration: 200 mg/kg DLI: 24 h Wavelength: 660 nm Energy: 1.25 W/cm^2^ Treatment time: 10 min	CCK-8 assay Flow cytometry CLSM assay	Tumor regression efficiency up to 80%, thus extending the survival rate
	In vivo			PS: PROTAC Concentration: 10.0 mg/kg DLI: 36 h Wavelength: 671 nm Light source: laser Energy: 400 mW/cm^2^ Treatment time: 5 min Treatment duration: 3 cycles at 3-day intervals	H&E staining TUNEL staining PCR	

**Legend:** DLI—drug-light interval; H&E—Haemotoxylin and Eosin; MTT—3-[4,5-dimethylthiazol-2-yl]-2,5 diphenyl tetrazolium bromide; PCR—polymerase chain reaction; TUNEL—Terminal deoxynucleotidyl transferase dUTP nick-end labeling.

**Table 4:** Studies addressing combination strategies.

Ref.	Model	Disease/Source of CSCs	Target/Targeting Strategy	Photodynamic Approach	Methodology for Assessing the Results	Main Results
[44]	In vitro	Human Breast cancer MCF-7 MDA-MB-231 cells	CD44 CD44 targeted with HA@DTX/TPCS_2a_-NPs	PS: TPCS_2a_ Concentration: 0.25 µg/mL DLI: 24 h Wavelength: 600–800 nm Light source: red light Energy: 1 J/cm^2^	ALDH activity assay and cellular uptake	Stemness and self-renewal capacity were severely hampered
[45]	In vitro	Human Breast cancer MDA-MB-231 MCF7 cells	CD44 HC60S-DI NPs	PS: C60 fullerene DLI: 12 h Light source: NIR light Energy: 1.5 W/cm^2^ Treatment time: 3 min	In vivo imaging Immunohistochemical staining Flow cytometry	High drug loading in NPs could target and inhibit breast CSC Tumor volume reduction
	In vivo			PS: C60 fullerene Concentration: 1.5 mg/kg DLI: 12 h Light source: NIR light Energy: 0.7 W/cm^2^ Treatment time: 3 min		
[46]	In vitro	Human ovarian cancer HEY-T30	CD44 PheoA-SN38-HC NPs	PS: PheoA Concentration: 1 mg/mL DLI: 4 h Wavelength: 670 nm Light source: NIR light Energy: 100 J/cm^2^	Western blot	High tumor damage, suggesting the synergetic effect of PDT/chemo combination therapy
	In vivo			PS: PheoA Concentration: 10 mg/kg Wavelength: 671 nm Light source: NIR light Energy: 100 J/cm^2^ Treatment duration: once a day for 3 days	H&E and TUNEL staining	
[47]	In vitro	Human Cervical carcinoma HeLa cells Mouse cervical carcinoma U14 cells	PDA@GNRs-DOX/Ce6	PS: Ce6 Concentration: 12 mg/kg DLI: first and sixth day, 24 h Wavelength: 650 nm Light source: xenon light Energy: 100 mW/cm^2^ Treatment time: 15 min Treatment duration:12 days	MTT assay Fluorescence microscope (2′,7′-dichlorofluorescein diacetate (DCFH-DA) probe)	Biocompatibility, cell shrinkage, nuclei deletion, and promote apoptosis Insignificant hepatic toxicity and decreased LDH levels
	In vivo				Photothermal camera Biochemical indexes (ocular blood serum) H&E assay	
[48]	In vitro	Human Breast cancer 4T1 cells	PMT	PS: TBP-2 Concentration: 10 µg/mL DLI: 2 h Light source: white light Energy: 0.1 W/cm^2^ Treatment time: 10 min	Viability assay Clonogenic assay DNA double-strand breaks	CSC marker expression decreased after treatment (ALDH and CD133)
	In vivo			PS: TBP-2 Concentration: 5 mg/kg DLI: 12 h Light source: white light Energy: 0.5 W/cm^2^ Treatment time: 10 min Treatment duration: two consecutive days	H&E staining TUNEL Ki67 staining	
[49]	In vitro	Human Undifferentiated colon carcinoma CT26 cells	Platelet cell membrane-coated TBP-2@TaOx	PS: TBP-2 Concentration: 50 µg/mL DLI: 2 h Light source: white light Energy: 0.5 W/cm^2^ Treatment time: 5 min	Clonogenic assay DNA double-strand breaks ROS levels	Eliminate tumor and reduce recurrence as well as side effects
	In vivo			PS: TBP-2 Concentration: 40 mg/kg DLI: 12 h Light source: white light Energy: 0.5 W/cm^2^ Treatment time: 5 min Treatment duration: once		
[21]	In vitro	Human Breast cancer 4T1 cells	ALDH PS-CHO@HA-ATRA-SS-ATRA	PS: PS-CHO Concentration: 20 µg/mL DLI: 4 h–3 days Light source: white light Energy: 40 mW/cm^2^ Treatment time: 4–5 min	ALDH expression by flow cytometry RNA extraction kit HIF-a by immunofluorescence	ATRA reduced stemness Inhibited tumor growth
	In vivo			PS: PS-CHO Concentration: 5 mg/kg DLI: 12 h Wavelength: n.d. Light source: white light Energy: 60 mW/cm^2^ Treatment time: 5 min	In vivo imaging	
[50]	In vitro	Human colon carcinoma HCT116 cells	MKCe6 NPs	PS: Ce6 Concentration: 6.5 µM DLI: 4 h Wavelength: 650 nm Light source: xenon light Energy: 7.5 mW/cm^2^ Treatment time: 20 min Treatment duration: first and third day	Transwell migration Wound healing RT-qPCR	Effective tumor growth repression and tumorigenesis inhibition
	In vivo				H&E staining Immunofluorescence Western blot	
[51]	In vitro	Human Breast cancer 4T1 cells	HP@PP/LP NPs	PS: Ce6 Concentration: 4 µg/mL DLI: 6 h Wavelength: 660 nm Light source: NIR laser Treatment time: 4 min	ROS levels EMT expression Migration assay Flow cytometry	Promotes BCSC differentiation, EMT blockage, as well as PDT effect Tumor growth inhibition
	In vivo			PS: Ce6 Concentration: 2.5 mg/kg DLI: 6 h Wavelength: 660 nm Light source: NIR laser Energy: 0.1 W/cm^2^ Treatment time: 10 min	Physiology changes	
[52]	In vitro	Human Breast cancer 4T1 cells	SEED NPs (ATRA/Ppa-TK-DOX NPs)	PS: Ppa Concentration: 5 μg/mL (Ppa-TK-DOX) and 2.5 μg/mL (ATRA) DLI: 24 h Wavelength: 660 nm Light source: laser Energy: 100 mW/cm^2^ Treatment time: 5–10 min	ROS levels Cell migration and invasion	CSC differentiation and turns CSC chemosensitive Downregulating stemness markers 4-fold increase in tumor-targeted accumulation
	In vivo				H&E and TUNEL staining Ki67 staining Immunohistochemical Immunofluorescence	
[53]	In vitro	Human Breast cancer 4T1 cells	M1-IR/AT@HPOC	PS: IR-780 Concentration: 1–15 μg/mL Wavelength: 808 nm Light source: NIR laser Energy: 1.5 W/cm^2^ Treatment time: 3 min	CKK-8 assay Live- and dead-cell staining Confocal microscopy ELISA assays H&E staining	CSC self-renewal inhibition, hyperthermia induction Boost of immunogenicity cell death Differentiation of CSCs in non-CSC- and ROS-level alteration
	In vivo			PS: IR-780 Concentration: 2.5 mg/kg DLI: 12 h Wavelength: 808 nm Light source: NIR laser Energy: 1.5 W/cm^2^ Treatment time: 5 min		
[54]	In vitro	Human Breast cancer MDA-MB-231 4T1 cells	COHF NPs	PS: CyOA Concentration: 1 µM Wavelength: 660 nm Energy: 100–200 mW/cm^2^ Treatment time: 2–10 min	NADP+/NADPH assay kit Cellular uptake Confocal laser Flow cytometry Transwell migration assay Western blot RT-qPCR	High phototoxicity Increased tumor apoptosis and necrosis
	In vivo			PS: CyOA Concentration: 10 µmol/kg DLI: 4 h after last injection Wavelength: 660 nm Energy: 200 mW/cm^2^ Treatment time: 10 min Treatment duration: 5 times in 2 days	Western blot Immunofluorescence staining and imaging	
[55]	In vitro	Human Breast cancer 4T1 cells	FLG/ZnPc	PS: ZnPc Concentration: 0 to 50 µg/mL DLI: 12 h Wavelength: 808 nm Energy: 2 W/cm^2^ Treatment time: 5 min	Immunofluorescence ROS levels	Break of crosstalk of tumor vasculature and CSC ROS-induced breast CSC differentiation Suppression of tumor proliferation
	In vivo			PS: ZnPc Concentration: 6 mg/kg DLI: 8 h and 24 h Wavelength: 671 nm Energy: 15 and 30 mW/cm^2^ Treatment time: 5 min Treatment duration: three times every two days (6 days)	Immunohistochemistry	
[56]	In vitro	Human Colorectal cancer HT29 cells	MnOx/PDA@M	PS: MnOx/PDA Concentration: 200 μg/mL DLI: 12 h Wavelength: 808 nm Light source: NIR light Energy: 2 W/cm^2^ Treatment time: 3 min	Trypan blue	Tumor inhibition rate is 70.8% in colorectal cancer Primary and secondary spheres are significantly reduced in number and size
	In vivo			PS: MnOx/PDA Concentration: 1 mg/mL DLI: 12 h Wavelength: 808 nm Light source: NIR light Energy: 2 W/cm^2^ Treatment time: 5 min	Photoacoustic imaging	

**Legend**: ALDH—Aldehyde dehydrogenase; ATRA—All-trans retinoic acid; CSC—Cancer stem cells; DLI—drug-light interval; DNA—deoxyribonucleic acid; EMT—epithelial-to-mesenchymal transition; H&E—Haemotoxylin and Eosin; HIF—Hypoxia-inducible factor; LDH—lactate dehydrogenase; MTT—3-[4,5-dimethylthiazol-2-yl]-2,5 diphenyl tetrazolium bromide; NADP+/NADPH—nicotinamide adenine dinucleotide phosphate; NIR—Near-Infrared; NPs—nanoparticles; TUNEL—Terminal deoxynucleotidyl transferase dUTP nick-end labeling.

## Data Availability

Data is contained within the article.

## Abbreviations

The following abbreviations are used in this manuscript:

CSCs	Cancer stem cells
ROS	Reactive oxygen species
HIF	Hypoxia-inducible factors
EMT	Epithelial-to-mesenchymal transition
DNA	Deoxyribonucleic acid
PDT	Photodynamic therapy
PRISMA-ScR	Preferred Reporting Items for Systematic Reviews and Meta-Analysis, extension for Scoping Reviews
DLI	Drug-light interval
ALDH	Aldehyde dehydrogenase
TF	Tissue factor
Lgr5	Leucine-rich repeat-containing G-protein-coupled receptor 5
AlPcS_4_Cl	Sulphonated Aluminum Phthalocyanine Chloride
SAL	Salinomycin
NIR	Near-infrared
TPCS_2_a	Meso-tetraphenyl chlorine disulfonate
DTX	Docetaxel
HA	Hyaluronan
DOX	Doxorubicin
PDA	Polydopamine
EPR	Enhanced permeability and retention
ATRA	All-trans retinoic acid
PCI	Photochemical internalization
